# War on Two Fronts: The Fight against Parasites in Korea and Vietnam

**DOI:** 10.1017/mdh.2017.35

**Published:** 2017-07

**Authors:** Mark Harrison, Sung Vin Yim

**Affiliations:** 1 Wellcome Unit for the History of Medicine, University of Oxford, 45-47 Banbury Road, Oxford OX2 6PE, UK; 2 Kyung Hee University College of Medicine, Seoul 130-701, Korea

**Keywords:** Vietnam War, Republic of Korea, Parasites, Malaria, Communism, Aid

## Abstract

The Vietnam War has long been regarded as pivotal in the history of the Republic of Korea, although its involvement in this conflict remains controversial. While most scholarship has focused on the political and economic ramifications of the war – and allegations of brutality by Korean troops – few scholars have considered the impact of the conflict upon medicine and public health. This article argues that the war had a transformative impact on medical careers and public health in Korea, and that this can be most clearly seen in efforts to control parasitic diseases. These diseases were a major drain on military manpower and a matter of growing concern domestically. The deployment to Vietnam boosted research into parasitic diseases of all kinds and accelerated the domestic campaign to control malaria and intestinal parasites. It also had a formative impact upon the development of overseas aid.

Between 1965 and 1973, the Republic of Korea (ROK) sent over 300 000 soldiers and tens of thousands of civilians to Vietnam in support of the American-led war against communism. The ROK was by no means unique in this respect, as United States forces also received military aid from Australia, New Zealand, the Philippines and Thailand. Civilian assistance for non-communist South Vietnam was provided by an even greater number of nations, including those of non-combatants such as the United Kingdom. But the South Korean deployment was by far the largest apart from that of the United States of America and was disproportionate to the country’s population, not to mention its prosperity. One of the reasons for this was that the ROK was in the front-line of a geopolitical struggle between capitalism and communism. As one semi-official magazine put it, a ‘Communist take-over of Vietnam would be a direct threat to the security of the Republic of Korea.’ However, the same publication identified other reasons for the Republic’s involvement. It would ‘enhance the international prestige of the Korean people by demonstrating their traditional chivalrous spirit’ and express their gratitude to nations which had supported their own struggle against communism.^1^ Finally, the magazine alluded to what many have since come to regard as the most important motives for the ROK’s intervention in Vietnam: the calculation that it would secure aid to modernise its economy and ensure America’s continuing commitment to its defence.^2^

While most historians conclude that the war allowed the ROK to achieve these objectives, some regard its role as akin to that of a ‘mercenary’, fighting an imperialistic war on behalf of the USA.^3^ The ROK’s war record has also been dogged by allegations of atrocities and sexual slavery, some of which only came to light after the war had ended.^4^ For their part, Korean veterans have reacted angrily to these claims and have protested about the inadequacy of financial compensation and medical treatment for those suffering from war-related health problems.^5^ It is not the purpose of this article to evaluate these claims, although they do have a bearing on what follows. Its aim is rather to examine an aspect of Korea’s wartime experience that has so far received little attention – the ways in which the war affected the development of medicine and public health.

That the Vietnam War made any appreciable impact on Korean medicine may be surprising but medicine figured prominently from the very beginning of Korea’s involvement. When the ROK was invited by the US and South Vietnam to send military assistance in January 1964, the government’s response was to dispatch a mobile surgical hospital rather than combat troops.^6^ Thereafter the number of medical units in Vietnam grew rapidly, as they accompanied divisions of infantry and marines. Medical teams were also sent to assist Vietnamese civilians. This large mobilisation transformed the careers of many of those involved and had far-reaching implications for medicine in the ROK.

One of the few historians to have recognised the significance of the war for Korean medicine is John P. DiMoia, who has noted that military requirements were critical to the growth of certain specialities, particularly parasitology.^7^ Building on his work, and that of other scholars of public health in independent Korea,^8^ this article will consider the ways in which military and civilian priorities intersected in relation to infectious disease. Focusing on parasitic diseases, which were the chief concern of the military at this time, it argues that the Vietnam War enlarged the scope of scientific enquiry and enabled Korean doctors to gain greater recognition internationally and at home. Although some specialised in particular diseases such as malaria, many worked on a variety of protozoal and intestinal infections, as well as other kinds of disease. It is only by considering their work across a fairly wide spectrum that we can hope to understand the impact of war on their careers. Secondly, the article argues that the conflict in Vietnam added momentum to public health campaigns waged simultaneously in the ROK. In both countries, Korean doctors came to believe that they were involved in similar struggles – against ‘ignorance’ and harmful traditions but pre-eminently against communism, in whatever guise it presented itself. These efforts focused on the eradication of parasites and by considering these diseases we may gain insights into the broader fields of infectious disease and preventive medicine.

Although armed conflict usually prioritises certain areas of health and medicine, wartime innovations tend to be enduring only insofar as they meet other needs.^9^ In order to understand how civil and military concerns became entangled in Korea, it is necessary to take stock of developments in the Republic in the years before and during the Vietnam War. This is the purpose of the first section of this article, which shows that progress in infectious disease control was somewhat slower in South Korea than in the North, casting doubt on the Republic’s capacity to deal with the threat of communism. The remaining sections examine the relationship of Korean medical practitioners to the war in Vietnam. The spotlight falls first on doctors who were able to boost their careers through research on malaria and intestinal parasites among ROK troops. This section shows how wartime priorities intensified anxieties over parasitic infections in the ROK, among civilians as well as soldiers. The third section of the article examines the role of the Korean preventive medicine team, KOPREM, and the relationship between its work in Vietnam and domestic concerns. Focusing particularly on the career of KOPREM’s leader, Dr Chu Jeong-Gyun, we can see how the war enabled specialists in infectious diseases to acquire greater status and influence. The final section develops these themes, showing how wartime propaganda accentuated the ROK’s medical role in Vietnam and boosted the reputation of its doctors at home and overseas.

## 1 Public Health in Independent Korea

Western medicine was introduced into Korea through two channels: the Japanese, who annexed Korea in 1910, and Western missionaries, most of whom were from the United States.^10^ Missionary doctors, colonial physicians and administrators placed a great deal of emphasis on hygiene and the prevalence of infectious diseases invited comment among both. Apart from epidemic diseases such as smallpox and cholera,^11^ there were numerous endemic infections including an enormous variety of parasitic diseases, the most common of which was malaria. Many adult Koreans had acquired immunity to malaria after exposure in their childhood but foreigners suffered badly.^12^ Nevertheless, the prevalence of malaria afforded opportunities for scientific investigation and some Japanese doctors worked on aspects of malaria epidemiology, entomology and therapeutics.^13^ Similar opportunities were presented by other parasitic infections, including insect-borne parasites such as the filarial worm (common on the southern island of Jeju); hookworm or ankylostomiasis (more or less ubiquitous in agricultural areas); and paragonimiasis, caused by the lung-fluke.^14^ Yet the Japanese did very little to combat malaria or, indeed, other parasitic diseases among the Koreans.^15^

When US forces occupied Korea after the Second World War, infectious diseases were again in the spotlight. The US Army Military Government, which lasted from September 1945 to August 1948, was imposed primarily to check the spread of communism, which entailed dismantling the short-lived People’s Committees and supporting elections sponsored by the United Nations. These actions divided the country between the South, under US control, and the communist North. This made the US administration unpopular, as did the reappointment of Japanese former colonial officials as advisors.^16^ Lacking deep-rooted support, and with an influx of refugees from the North, US-controlled Korea was chaotic and diseases spread easily. The Americans gave priority to those – like cholera – that had the potential to provoke civil disorder or harm US troops.^17^ However, they were also concerned about the prevalence of endemic infections, some of which debilitated their forces. Chief among these was malaria, which had become more widespread during the Second World War. Although the form of malaria most prevalent in Korea (*Plasmodium vivax*) was less deadly than that responsible for military deaths in the Pacific War (*Plasmodium falciparum*), morbidity among American forces was huge. A concerted effort to control malaria through the use of DDT and the administration of anti-malarial drugs began in 1945 but the benefits to Koreans were limited because efforts were concentrated in urban areas and around American bases.^18^

Like the Japanese before them, American doctors also remarked on the prevalence of intestinal parasites such as the large roundworm *Ascaris lumbricoides*.^19^ This parasite thrived in Korea because human excrement was practically the only method used to fertilise the land, possibly due to the relative scarcity of cattle. However, American doctors believed it would be difficult to change customs that had been ‘in vogue for centuries’.^20^ The problem was aggravated by the failure to detect and treat many of those who were infested. As H.F. Smith of the US Public Health Service put it, ‘sick Koreans rarely enjoy the privilege of medical attention or hospitalisation’, more sick individuals being ‘treated by Han-ei [*sic*: Han-ui] practitioners (herb doctors) than by modern physicians’.^21^ Parasitic infection was assumed to be ‘practically universal’.^22^

The war of 1950–3 inevitably increased mortality and morbidity throughout the Korean peninsula but the return to more stable conditions brought gradual improvement. Mortality fell from a high of 33 per 1000 in 1950–5 to 16.1 per 1000 in 1955–60.^23^ But progress made north of the border with Soviet assistance was even more impressive. In 1960, the crude death rate in the Democratic People’s Republic of Korea (DPRK) was lower than in the ROK and probably remained so for the rest of the decade.^24^ Specialists in preventive medicine attributed the ROK’s slower progress to a combination of ‘outdated’ ideas about how to control infectious disease and the inadequacy of medical provisions. Another target was *Han Yak*, or Korean traditional medicine, which prescribed remedies such as eels, raw snake-meat and children’s urine. These ‘ineffective and ludicrous’ treatments, as they were described by some practitioners of Western medicine, were prevalent throughout the country and it was difficult to counter them due to the lack of modern facilities.^25^

The balance began to tilt in favour of biomedicine after Park Chung-Hee became president in 1962, following a military coup the previous year. A healthy workforce and military were essential to Park’s drive for economic and military modernisation.^26^ Park may have also regarded the improvement of health as a way of legitimising his authoritarian regime.^27^ As well as introducing Korea’s first national insurance scheme in 1963, he took several steps to reduce the burden of disease, including his decision in 1965 to increase the number of clinics for tuberculosis.^28^ Park also began to tackle another stubborn health problem – malaria – extending an initiative begun under President Lee Seung-Man (Rhee Syngman) in April 1959, with assistance from the World Health Organisation. The ‘malaria project’, as it was termed, made epidemiological information a priority and in 1960 malaria became a notifiable disease. Surveys were conducted to ascertain spleen rates and blood-parasite levels and these were augmented with public education and passive case detection.^29^ Later, a major public health campaign was orchestrated, utilising indoor residual spraying with DDT; the most effective way to apply the insecticide. In 1962, the ROK/WHO project was confidently renamed the ‘Malaria Pre-Eradication Programme’ and, by the time of the Korean deployment to Vietnam, some areas of the country had been declared free from the disease.^30^

When the Americans arrived in Korea in 1945, their lack of local knowledge and the shortage of indigenous medical personnel induced them to employ Japanese who had worked in Korea as colonial officials.^31^ These appointments were strongly and sometimes successfully resisted by Koreans, who resented what seemed like a return to colonial rule. But over the next few decades, many Koreans acquired expertise in a range of medical disciplines, including those related to infectious diseases. Infectious disease doctors regarded it as their duty to improve public health through more vigorous policies and to dispel public ignorance. There was particular interest in parasitic infections, for their prevalence had been regarded by the Japanese and the Americans as symptomatic of Korea’s backwardness. By reducing the amount of parasitic infection, Korean physicians hoped to rid their country of the stigma it had carried for so long.^32^

In addition to work on malaria, there were numerous studies of the distribution of intestinal parasites, many of which ascribed infection to the living conditions and dietary habits of the population, particularly the rural poor. A typical example was the investigation undertaken by Professor Kang of Seoul National University, which examined 461 school students in the Gimhae area between September 1951 and May 1952. Seven types of intestinal parasite were found among the sample population. Some 89% of the children were found to be infected with *Ascaris lumbricoides* and 41% with hookworm, both of which were linked to insanitary conditions. *Clonorchis sinensis* (the liver fluke) was present in 32% – an infection associated with the consumption of uncooked fish and shellfish. The growth of infected children was found to be significantly impaired compared with those who were not infected.^33^

The perceived importance of parasitology to national development was recognised by the creation of specialised departments in all the leading medical schools.^34^ In addition, in 1959, the Korean Society of Parasitology was established and began to hold annual conferences. Its proceedings and original research articles were published in the Society’s journal, the *Korean Journal of Parasitology*, from 1963. However, parasitology was making its presence felt in other fields of medicine. In 1960, the *Journal of Pediatrics* (an American publication) carried an article on parasites among children in Korea, this time on the lung-fluke infection paragonimiasis, which was thought to be more prevalent in Korea than in any other country. The author, Dr Yun, of Yonsei University’s Severance Hospital, claimed that the incidence of this infection was often related to the consumption of raw crayfish, sometimes in the mistaken belief that it cured measles. Yun recommended an education campaign to deter the practice.^35^ Paragonimiasis was studied at other hospitals, too. In the Presbyterian Hospital at Daegu, Park Choong-Moo conducted a survey among patients in his paediatric ward and found the disease to be endemic among them. He again noted the prevalence of dangerous customs, including consumption of the juice of crushed crabs for colds and coughs, and the juice of crayfish for measles – practices that spread widely across Korea during the war of 1950–3. The risks presented by such habits were also communicated in newspapers, which summarised the latest research for a popular readership.^36^ However, some doctors were beginning to doubt whether education alone would be adequate. Researchers from Yonsei University’s Department of Parasitology argued that faecal contamination of food was so widespread that more drastic action needed to be taken.^37^ They therefore recommended that human faeces should be sterilised to prevent cysts and ova being spread by insect vectors and other means after it was scattered as manure.^38^

In 1964, the Korea Association of Parasite Eradication (KAPE) was formed to galvanise these efforts, with the goal of reaching a zero rate of infection within ten years.^39^ But the government was not yet prepared to finance a scheme as ambitious as that proposed by the KAPE. The campaign against intestinal parasites therefore lagged behind those to eradicate tuberculosis and malaria, both of which had received financial support; not only from the government of the ROK but also, in the case of malaria, from the United States. Furthermore, Koreans showed little sign of changing their ways. Many still preferred traditional medicine, which had been granted official recognition alongside Western medicine in 1951.^40^ Despite growing competition from their biomedical equivalents, traditional doctors remained prosperous and influential.^41^

## 2 Medicine and the Military

When the first Korean medical teams were dispatched to Vietnam, they encountered a similarly pluralistic environment. Western medicine had failed to make much headway despite being endorsed by the French colonial administration.^42^ The medical situation also resembled that in Korea, inasmuch as infectious diseases were rife and sanitary conditions in many towns and villages were poor. Korean doctors hoped that victory over disease in such a setting would provide an object lesson in the superiority of scientific medicine and the organisational capacity of the ‘Free World’ – the term used by the US and its allies to describe capitalist states, particularly those antagonistic to communism. The Koreans worked alongside medical teams not only from combatant nations but also non-combatant ones such as West Germany.^43^ The governments of these countries had concerns about the progress of communism and viewed aid to civilians as a way of winning hearts and minds. Doctors from the ROK also believed that success in Vietnam would enable them to secure victory over similar forces at home. As they saw it, they were fighting a war on two fronts – against the combined foes of ignorance, arrested development and communism.

The majority of doctors who served in Vietnam did so in the armed forces. Although there were some long-serving professionals, most were conscripts, conscription having existed in the ROK since 1949. However, compulsory service did little to weaken the commitment of Korean soldiers. The experience of fighting in Vietnam tended to intensify patriotic feelings and reinforced convictions about the struggle against communism. Korean troops subsequently became known for their ideological zeal; something that was relatively rare among American conscripts.^44^ On these matters, there is no indication that military doctors felt different from those in combatant roles. The same is true of civilian doctors and clinicians who joined their military colleagues in Vietnam. Most were proud of their achievements during the war and worked tirelessly for the cause. Prominent among their work was the treatment, prevention and epidemiology of parasitic infections. Some doctors specialised in particular diseases but most worked on a range of infections including malaria and intestinal parasites. Their wartime contributions did much to advance their careers and, like American doctors who were drafted contemporaneously, the training and networking opportunities presented by military service assisted their rise to leadership in civilian life.^45^ However, in these respects, the war in Vietnam was far from unique, for the exigencies of armed conflict have often enabled medical specialists to acquire authority and prestige.^46^

One of the biggest problems for military doctors in Vietnam was malaria, which plagued Korean troops as much as those of other nations.^47^ One of their first priorities was to determine the nature and extent of malarial infection among ROK soldiers and some of the most important contributions in the first years of the deployment came from Kim Doo-Hie, a preventive medicine specialist attached to the Medical General Laboratory of the army. Kim wrote three articles on malaria, published in the *Journal of the Korean Medical Association* between 1967 and 1969. His first study focused on epidemiology, examining the relationship between malaria cases and temperature and rainfall, as well as prevalence rates in units dispersed across Vietnam. One interesting feature of this study is that it shows the incidence of malaria among different ranks and according to the nature of duties performed in the three months before sickness appeared. Incidence was highest among the lowest ranks (57% of all cases were among privates and 17% cent among corporals) and among infantry (34% of cases were rifle-men and 11% squad leaders).^48^ This much was predictable, as troops in forward areas were more exposed to human reservoirs of infection and malaria-bearing mosquitoes. But the variance between these groups was higher than expected, as Korean troops had been directed to take one tablet of chloroquine weekly as a prophylactic, as well as to use insect repellents and mosquito nets. In theory, these measures should have kept the incidence of malaria low, while reducing differences in morbidity between units. Despite these precautions, incidence remained high, at 111 per 1000, and varied sharply according to the area in which the troops were deployed. Kim concluded that this was the result of the infrequent use of mosquito nets and the failure to take malaria pills at the prescribed dosage.^49^ In fact, doctors of all nationalities tended to assume that most cases of malaria were due to lapses in discipline.^50^

After the publication of Kim’s research, it became apparent that the situation was more complicated than he had thought. His subsequent research revealed that well over 80% of soldiers questioned were taking the correct dosage of drugs at regular intervals. Despite this, there was still an extremely high rate of infection: 454 per 1000 troops. These findings accord with accounts by senior army officers such as Gen. Chae Myeong-Shin, the commander-in-chief of Korean forces in Vietnam, who recalled that anti-malaria measures were enforced rigorously.^51^ The only reasonable inference was that the drugs were losing their potency and ROK forces therefore began to use chloroquine in combination with other drugs. One of those being considered was dapsone, which had hitherto been used to treat bacterial infections. At the beginning of the war, there were dapsone trials among prisoners in the United States and, in 1966, among US soldiers in Vietnam. The ROK followed the US example but while the American results were initially encouraging, the Koreans found no clear advantage.^52^ Results obtained from trials of other drug combinations were more promising.^53^ As a result, dapsone was not widely employed as a suppressive treatment by ROK forces, unlike those of the US, where it continued to be used in combination with quinine and pyrimethamine.^54^

The Korean results were subsequently confirmed by the Australian Army, which began trials of dapsone in 1968. Australian doctors found they could not distinguish the drug’s potentially beneficial effects from epidemiological factors such as distance from reservoirs of infection. Moreover, the drug often produced harmful side-effects.^55^ Put simply, there was more chance of a soldier dying from dapsone than from malaria. Dapsone was therefore taken out of use in the Australian Army once troops were moved from high risk areas. This decision validated the approach taken by the Koreans but they needed no encouragement to depart quickly and decisively from the course of treatment taken by their senior partner, the US. Their independence in this matter indicates both the self-confidence of Korean doctors and the Army’s confidence in them.

The high reputation earned by Korean military doctors in Vietnam served them well in civilian life, as is shown by the subsequent career of Kim Doo-Hie. His second study on malaria was published just after he had left the army, having returned to his position in the Department of Preventive Medicine at Kyungpook (Kyungbuk) University in Daegu. There, he wrote the third of a series of articles on Vietnam, this one being entomological in nature, examining the distribution of mosquito species in Vietnam. The latter study represented the fruits of collaboration with several other scientists: two others from the Department of Preventive Medicine and Public Health at Kyungbuk plus Yang Kyung-Ho of the Surgeon-General’s Office of the ROK Army and Lee Kyu-Dong of the 33rd Army Hospital.^56^ Its mixed authorship demonstrates the porous boundary that then existed between military and civilian spheres.^57^ Most civilian doctors had previously served in the armed forces and this made co-operation easy, especially as there was a common national goal. Furthermore, military research was regarded as applicable to civilian contexts. It was partly for this reason that Kim combined his three articles on Vietnam with a number of papers on civilian public health to form a doctoral thesis. In it, he argued that military experience was transferable to the prevention of infectious diseases at home, whether it concerned the organisation of health campaigns or specific techniques of prevention.^58^

Kim published on aspects of his Vietnam research for several more years, notably on the problem of detecting asymptomatic cases of malaria.^59^ Although his work covered a wide spectrum of diseases including tuberculosis and typhoid, it is notable that he earned his reputation by working on infections deemed to be of the highest national importance. In this respect, he was by no means alone, as we can see from the career of Choi Sam-Sup, of the Department of Preventive Medicine at Chonnam (Jeonnam) University, in Gwang-Ju city. Choi directed one of the trials on suppressive treatment for malaria, mentioned earlier, but he also made an important epidemiological study of ROK forces in Vietnam. Like Kim, he found that the incidence of malaria differed considerably from one area to another, depending on the density of malarial infection among the local population, the duration of exposure to malaria vectors, seasonality, and the attitude of troops towards prophylactic measures. He also highlighted the persistence of malarial infection among those returning from Vietnam but whereas Kim had simply warned of this problem, Choi provided hard figures, finding that 2.4 per 1000 persons who had returned to Korea subsequently presented as malaria cases.^60^ Similar studies on returnees were conducted afterwards and enabled the problem to be fully understood and acted upon.^61^ Reinfection of parts of Korea from which malaria had been eradicated was thereby averted.

Having taken the lead in this research, Choi acquired a high reputation as an epidemiologist, on the strength of which he obtained a new position at the Department of Preventive Medicine at Ewha Women’s University, in Seoul. There, he continued to conduct epidemiological research (into various parasitic and non-parasitic diseases), as well as taking an active role in medical education. Through his work in these areas, he became a senior figure in the Korean Medical Association.^62^ Choi’s rise to prominence provides further evidence that wartime experience enhanced the careers of those who worked as specialists in preventive medicine and infectious disease.

The studies conducted by the likes of Kim and Choi were important because they had clinical as well as preventive implications, being useful in devising courses of treatment for returnees and soldiers who remained in Vietnam. There was also a good deal of purely clinical research undertaken at this time, in large hospitals in Korea as well as in evacuation facilities closer to the fighting. The purpose of these studies ranged from charting the clinical picture of malaria as suffered by Korean troops;^63^ severe complications such as ‘blackwater fever’, in which red blood cells burst and release haemoglobin directly into the blood vessels and urine;^64^ and, most importantly, the problem of resistance to the drug chloroquine.^65^ Further clinical research showed the combinations of drugs that worked best for different kinds of malaria.^66^ Apart from its immediate practical value, this research was important because it marked the penetration of parasitology into clinical medicine – the branch of Western medicine that had the highest status in Korea. Articles on the treatment of malaria were now as likely to appear in mainstream journals as in more specialist publications.

Korean doctors also made use of their time in Vietnam to work on intestinal parasites, the high incidence of which among ROK forces was considered shameful as well as injurious to manpower.^67^ One wide-ranging study was carried out by researchers from the Department of Parasitology at Seoul National University, who conducted a comparative examination of parasite levels in ROK troops in Vietnam, US forces and Vietnamese civilians, using soldiers based in Korea as a control group. Although some types of parasite (e.g. roundworms) were less common among ROK troops in Vietnam than among Vietnamese civilians, in most cases the pattern was reversed. While the overall rate of parasitic infection among Korean troops in Vietnam was lower than soldiers at home, it was substantially higher than among Vietnamese civilians. Over 82% of Korean soldiers in Vietnam were found to have some form of parasite, often several kinds, whereas the percentage of Vietnamese civilians infected was 64.6%. The infection rate for all parasites among US forces was only 21%.^68^ These findings were subsequently confirmed by others undertaken in the ROK. In one, 95% of the sample of 1012 soldiers were shown to be infected by one or more parasites.^69^ Another study by scientists at the Army’s Medical General Laboratory, undertaken from April 1970 to December 1971, showed only a marginal improvement, with 89% found to be infected.^70^

One notable feature of this research is that some of the authors had earlier worked on malaria. For example, Drs Yoon, Lee and Seo were among the co-authors of a two-part study of parasites in ROK forces; the first part of which concentrated on malaria and the second on intestinal parasites. They also wrote other papers on both types of parasitic disease. Such versatility was a hallmark of Korean medicine at this time, reflecting the urgency of the wartime situation and the relatively small pool of expertise that then existed in the ROK. While their work on malaria helped to preserve gains that had been made in Korea before and during the war, their studies on intestinal parasites showed just how little progress had been made, even by comparison with less developed countries such as Vietnam. As a result, the problem of intestinal parasitic infection began to receive more attention in the ROK and specialists in the field became more prominent within the medical profession, as the number and distribution of their publications shows. This eventually resulted in more support from the government but an effective solution to the problem of parasites would emerge only gradually, not only from the experiences of military doctors but from those who tackled the equally difficult problem of health among Vietnamese civilians.

## 3 Pacification

Korean medical aid to Vietnamese civilians was part of the Free World Assistance Program and comprised curative medical and surgical teams (beyond the scope of this article), as well as specialists in preventive medicine. Most of the latter were in Vietnam for relatively short periods to undertake research on disease and devise preventive measures for Korean civilian contractors. The only organisation sent to assist the Vietnamese administration specifically was KOPREM, on whose activities we shall focus in this section.^71^ KOPREM was present in Vietnam from July 1967 to July 1970 and had its headquarters at Bình Hòa airbase, which was located not far to the north of Saigon, in what was known as Region III. This territory comprised eleven provinces around Saigon, excluding the capital. It lay in the fertile Mekong Delta and covered some 29 300 square kilometres, about 60% of which was cultivated land (rice paddies, corn fields and rubber plantations), 30% forest, and the remainder classified as wasteland. The population of Region III was estimated at 3 040 400 people, most of whom were located in cultivated areas; about seventy per cent were ethnically Vietnamese and the rest were Chinese, Cambodian and Montagnard (indigenous mountain tribes). Population density was relatively low (104 persons per square kilometre), being less than half of that of Vietnam as a whole. Although some of the population had received basic education, many children were illiterate because of wartime disruption of schooling.^72^

These were unpromising circumstances in which to conduct public health work, especially for a small team like KOPREM, which initially comprised three doctors, two sanitarians, two technicians and an administrative assistant. They were given an almost impossibly wide range of duties, including advising the Vietnamese administration, conducting epidemiological surveys, organising quarantines, examining water supplies and educating Vietnamese civilians.^73^ None of these tasks were made easier by the fact that the team were unable to speak Vietnamese and were reliant on interpreters throughout their time in Vietnam.^74^ Nevertheless, the Koreans saw their deployment as an opportunity to spread the twin gospels of hygiene and anti-communism, principles espoused messianically by KOPREM’s leader, Dr Chu Jung-Kyun (Jeong-Gyun).

Chu was appointed leader of KOPREM on account of his extensive experience in preventive medicine, in the Army and in civilian life. Having graduated from Dental Medical School in Japan in 1943 and, in 1947, in medicine, from Seoul National University, he afterwards worked at the Institute of Infectious Disease Prevention. In 1949, he joined the ROK Army as a Captain and taught at the Namsan Military School and the Army Medical School before the outbreak of the Korean War. During the war, he served with distinction, receiving the Order of Military Merit in 1952. Chu remained in the Army after the war, working at the Office of the Surgeon General from 1954 and at the Central Pathology Institute of the Army from 1957. He left the Army with the rank of Colonel in 1961 and was appointed Professor at Busan National University Medical School, where he later established the Department of Parasitology.^75^ In 1963, Chu contributed two articles to the first issue of the *Journal of Korean Parasitology*; one on the subject of liver fluke, the other on roundworms.^76^ In the coming years, he wrote articles on a variety of parasitic diseases but his work continued to embrace non-parasitic infections as well as general aspects of preventive medicine like health education. Such adaptability was typical of Korean doctors who worked in the fields of infectious disease and public health at this time.

At the beginning of 1967, Chu was working as a civilian consultant to the ROK Army in infectious disease prevention but his appointment as head of KOPREM soon took precedence and it was for that reason that he was dispatched to Vietnam. The situation confronting him was not dissimilar to that in Korea; or at least the Korea of recent memory. Diseases of many kinds were rife and the reporting of cases was sporadic. One of his most pressing tasks was therefore to gather epidemiological intelligence.^77^ But the Koreans faced difficulty in obtaining timely and reliable information, which Chu attributed to the interruption of communications by the war, apathy and the lack of Vietnamese health workers. The only accurate reports were apparently those submitted by Free World (chiefly American, Australian, Filipino and Korean) medical teams. Consequently, it was difficult to implement effective measures. According to Chu, if the Vietnamese reported an outbreak, they usually did so by letter rather than radio. By the time the Korean team arrived, the patients had often been discharged from hospital or had died.^78^ Chu believed that these deficiencies were the product of years of neglect and what he regarded as unchallenged ignorance, exemplified by the overwhelming preference of the Vietnamese for ‘Chinese’ (i.e. traditional) as opposed to Western medicine – about which many harboured suspicion.^79^ As a result of their preferences, the Vietnamese rarely visited modern medical facilities, except as a last resort.^80^ Chu claimed that they followed the orders of traditional doctors unquestioningly and even if their condition worsened, they did not blame the doctors but rather apologised to them.^81^ He stressed that this behaviour was the result of habit rather than necessity. Although many hospitals were under-staffed as a result of the conscription of doctors into the military, medical facilities were far from negligible and all charges for treatment – including hospital stays – were borne by the government.^82^ ‘The primary fact’, he insisted, ‘is that Chinese medicine is admired by the people and enjoys their confidence and greater prosperity enables the population to pay for it.’^83^

A gulf of understanding clearly existed between members of KOPREM and the Vietnamese villagers they were supposed to be assisting and this was aggravated by the shortage of local health workers, which Chu again blamed on the French.^84^ The colonial administration had been forced to accept a pluralistic system of medicine because of the expense and practical difficulties involved in promoting Western ideas and treatments, not to mention the nationalists’ attempts to revive local traditions.^85^ But they had not entirely given up on their mission to ‘modernise’ the Vietnamese. Although they had learned to adapt to Vietnamese realities,^86^ the French persisted in their attempts to improve public health, albeit with little obvious effect.^87^ But Chu appears to have been unaware of or was simply unimpressed by these efforts. His preference was distinctly for the American way in public health, most likely because of the formative role the US had played in post-war Korea. In 1946, the US military government undertook a major reform of higher education in which medical colleges were required to follow a US-style six-year curriculum and two state colleges (one from the former Japanese imperial university in Seoul) were combined to form the medical school of the newly established Seoul National University, which Chu attended.^88^ Although he graduated too early to benefit fully from the reformed curriculum, he was impressed by the resolve of the Americans and shared their commitment to fighting communism. Chu was appalled by the savagery of the Viet Cong and saddened and frustrated by the division of his own country. He viewed the campaign against disease in Korea and in Vietnam as necessary for the defeat of what he regarded as an existential threat.^89^

KOPREM initially directed most of its attention to epidemics of cholera and plague, in accordance with the programme devised by the Joint Preventive Medicine Subcommittee of the US-led Medical Policy and Coordinating Committee. The JPMS planned public health assistance for the Vietnamese administration and proposed that diseases causing the most disruption should be attacked first. Its report concluded that ‘The key to most rapid military, political and civil success is delivering essential opportunities for a better life to the mass of the people.’^90^ Tackling epidemics was only the first step towards this goal but it was an urgently necessary one, in view of the panic they created.

The same principles were behind US aid to Korea in the 1940s and to South Vietnam prior to US military involvement. From the mid-1950s, the US had enlisted the international community, including the World Health Organisation, in what was, essentially, a counter-insurgency campaign.^91^ These efforts gathered momentum after 1968, when a more ‘disciplined and scientific approach’ was taken to what was termed the ‘pacification’ of the Vietnamese people.^92^ The aim of pacification, as agreed between President Lyndon B. Johnson and Vietnamese officials in 1966, was to isolate villagers from communist insurgents. It was overseen by a body known as CORDS (Civil Operations and Revolutionary Development Support), which had an American military commander and a Vietnamese, civilian deputy. CORDS co-ordinated the efforts of both governments and various other civilian agencies, including those sent by other countries including the ROK.^93^ The work directed by CORDS was conceived as a form of psychological warfare.^94^ But PSYOPS extended far beyond the remit of CORDS, as it entailed collaboration with other constituencies such as the media. The media’s co-operation was deemed essential in view of the need to engage not only the Vietnamese but also audiences in countries sending aid and troops. As the body-count mounted, and as reports of Vietnamese civilian casualties appeared with greater frequency, publicising the humanitarian mission in Vietnam became more important.

Rapid results were essential in order to convince Vietnamese civilians that they were better off under the US-backed administration than under the communists.^95^ But disease control measures would need to be carefully explained rather than simply imposed, as they had often been in the past. As the JPMS warned, ‘The primary effort should be to make the people aware of the meaning of the disease control measures to their own health and welfare, and, at the same time, make the control measures attainable by the people.’^96^ This was particularly true of measures dealing with epidemic disease, which were likely to entail restrictions on movement or infringements of customary liberties and traditions. Although resistance of various kinds was evident in the case of measures introduced to deal with plague, Korean and other foreign medical teams succeeded in getting the disease under control.^97^

Other diseases proved more stubborn opponents and this was particularly true of malaria. When it was not involved in anti-epidemic activities, KOPREM devoted much of its attention to this disease, which Chu considered ‘one of the most important factors of morbidity and mortality in South Vietnam’.^98^ The situation there was similar to that which had existed in Korea some years previously but in South East Asia malaria presented in more diverse and deadly forms. As well as vivax malaria, which was familiar to most Koreans,^99^ most of the cases in Vietnam were caused by the falciparum parasite, which could easily cause fatalities among unprotected persons. In its efforts to control malaria, the Korean team was able to build on the work of the Vietnamese government, which had launched an eradication campaign in 1958.^100^ This had actively involved villagers, who were said to be appreciative of the protection afforded by eradication.^101^ But war altered the situation drastically: malaria was resurgent or epidemic in many areas, especially in towns where there had been mass migration from territory captured by the Viet Cong. Control measures focused on the routine spraying of homes with insecticides such as DDT but this method faltered as mosquitoes became resistant.^102^ In addition, drug resistance in cases of *Plasmodium falciparum* was spreading rapidly among civilians as well as soldiers.^103^ According to Chu, this was due to the lack of consistency in malaria chemotherapy. In Vietnamese hospitals, there was apparently ‘no conformity as to the type of drug used, the dosage employed nor the duration of the treatment’.^104^

Chu acknowledged that these failings resulted from wartime conditions but he claimed that his efforts to improve the situation were hampered by the lack of counterpart staff in the Vietnamese administration and their unwillingness to change their ways of working.^105^ This was deeply frustrating for Chu as he had entertained hopes that the Korean team would leave a lasting impression on Vietnam, by helping the Republic to build self-reliant communities inimical to socialism.^106^ For anti-communists like Chu, this was a very personal goal and he was particularly keen to contribute to what the JPMS saw as the second stage of its campaign against disease in Vietnam. While the first had focused on the chief epidemic diseases, the second entailed a protracted struggle against endemic infections, poverty and ‘ignorance’. As the JPMS put it in its initial report:

Health education is required primarily to fix the need for health and the means to health in the minds of the people. This is most effectively done through children in the schools. However, it can be part of the Revolutionary Development Program …. Immediate good will is often lost unless prolonged through snowball application to the people’s self-interest. PSYOPS is a resource that might be employed to advantage in the method of presentation of health education material.^107^

To this end, specialist units of the US Army were directed to work with international medical teams to tackle a range of infectious diseases, including those which posed no direct or immediate threat to military interests. Such interventions were not unprecedented but the use of psychological techniques had become more prominent.^108^ In Vietnam, they were widely employed in schools and in attempts to promote inoculation and other public health programmes among adults.^109^ Such efforts required close co-ordination between foreign medical teams and the civilian administration. As the Vietnamese appeared to lack basic knowledge about how to conduct public health campaigns, Chu insisted that it was ‘urgent to educate them to the attitudes of the Americans in regard to preventive medicine activities’.^110^ He also thought it necessary to instruct the public as to what they should expect from the civil authorities. As Chu pointed out in his first end of tour report, the ‘serving period of the Koprem is limited and the team will eventually leave when this country has settle[d] down’.^111^ His aim was not simply to contribute to the war effort – which he assumed would culminate in the defeat of communism – but to assist in the construction of a free and prosperous nation; in essence, to reprise the United States’ role in post-war Korea.

KOPREM thus established a pilot health education programme to complement its preventive activities, intended primarily for Vietnamese health workers such as sanitarians, midwives and village headmen.^112^ From late 1967, Chu and other members of his team moved from one district to another every few days, training between 20 and 60 health workers at a time.^113^ These methods were similar to those which had been used successfully in Korea, particularly the field training and village education programmes conducted as part of the malaria eradication programme.^114^ They continued until KOPREM was removed from Vietnam in 1970, for reasons which are unclear. It may be that the team was unable to make much progress with its educational work or simply that its members wished to return to the ROK, having spent much longer in Vietnam than most of their civilian colleagues. Another possibility is that KOPREM was removed because of growing disquiet over the US decision, in 1969, gradually to withdraw its troops from the country. However, the most likely reason is that Chu concluded that his expertise was required to deal with parasitic infection in his own country; a problem that had been highlighted by the war and which the government was now more determined to tackle.

## 4 Propaganda

Whatever the reasons for KOPREM’s withdrawal, it is clear that it had worked tirelessly in pursuance of the objectives it had been set. It could even claim some definite successes, most notably in its contributions to the fight against epidemics. But the broader goal of pacification had failed, such efforts amounting to ‘too little, too late’ in the view of one of its historians.^115^ In addition, health workers and villagers who collaborated with foreign teams were attacked by the Viet Cong and this naturally deterred many people from co-operating.^116^ Yet, medical assistance served other purposes rather better. One of its aims was to present a positive image of Korean involvement to allied nations and the public at home, and this became more important as rumours of misconduct by Korean troops began to circulate. In his end of tour report, Lt.-Gen. A.S. Collins Jr. wrote that in

every province and district senior advisors complain to me about ROK indifference to the concerns of the province and district chiefs. We received many hints from the Vietnamese that the ROKs had committed atrocities. Only a few of these allegations were clearly supported. However, there were so many instances of questionable actions that there must have been some basic disregard of the rights of the Vietnamese by the ROK soldiers.

Collins went on to complain that ROK soldiers were notorious for their ‘sharp practices in black market activities, currency manipulations, and supply operations’, adding that ‘This notoriety was well earned.’^117^ Some historians have even claimed that these atrocities were tolerated and encouraged by the Korean government.^118^

At any rate, the ROK’s humanitarian work among the Vietnamese was useful in distracting attention from reports of brutality by its soldiers. In 1968, for example, the Vietnamese magazine *Alliance for Freedom* lauded Korea’s commitment to medical, agricultural and construction programmes, noting that over 10 000 engineers had been dispatched to Vietnam (the number later increased to 23 000). The magazine concluded that Koreans were playing a vital part in the ‘Psychological War’ in Vietnam, remarking correctly that the ROK government considered it to be ‘the second anti-Communist front’.^119^ Although English-language newspapers such as *Alliance for Freedom* reached relatively few Vietnamese, they flattered and reassured the Republic’s supporters. Their message was reinforced by various official publications in the ROK, some written in English (presumably for American readers) as well as in Korean. They showed troops rescuing children in the heat of battle; providing medical aid to civilians; helping Vietnamese women with their harvests; handing out clean drinking water and delivering rice to the needy.^120^ The work of civilian teams in Vietnam was also mentioned; particularly in the spheres of health, agriculture and engineering. The aim of this propaganda was not simply to present a humanitarian image but to bolster confidence in a positive outcome to the war. Koreans wanted to know that their contribution was making a difference and in this, too, they were reassured, being informed that ‘civic medical treatment activities play an important role in winning the friendship and cooperation of the Vietnamese people, without which no ultimate victory in the war can be expected’.^121^

Official and semi-official accounts of military involvement in Vietnam were reinforced in Korean newspapers, most of which took a pro-government line. Typical in this respect were the Seoul-based newspapers the *Donga Ilbo* and *Chosun Ilbo*, popular dailies with a nationwide readership. These newspapers gave up-beat accounts of the military campaign, accompanied by images of Korean soldiers performing humanitarian acts. Articles and photos depicted the soldiers’ kindness to Vietnamese civilians and even to animals such as stray dogs.^122^ They stressed that every effort was being made to avoid civilian casualties.^123^ Civilian medical efforts, for instance campaigns against infectious disease, were also reported.^124^ Articles focused initially on the prevention of the major epidemics but increasingly on the prevalence of endemic infections such as those caused by parasites. Korean doctors were portrayed as ‘fighting for civilisation’ against ‘unbelievably strange customs’, although the similarity between some Vietnamese practices and those in rural Korea was acknowledged.^125^ Like their Korean counterparts, Vietnamese villagers were said to lack any concept of hygiene or of the ways in which parasites were transmitted. Seeking guidance, they looked to Korean medical workers like a child to its parents.^126^ These paternalistic messages were intended to reinforce convictions in the rightness and necessity of Korea’s intervention in Vietnam as well as the importance of similar campaigns at home. But, from the middle of 1968, more pessimistic reports began to appear from Vietnam. Korean involvement was still presented in a heroic light but it seemed that the ROK was being let down by its allies. There were reports of communication difficulties with American units as well as logistical failures.^127^ Moreover, from 1969 there was great unease about the prospect of US withdrawal from Vietnam and the policy of ‘Vietnamisation’ attendant upon it. By comparison with Korean troops, the morale and efficiency of Vietnamese republican forces was said to be poor.^128^

The eventual victory of communist forces in Vietnam represented a failure for the Republic of Korea but the country’s involvement in the war enabled it to secure the assistance it needed to modernise its economy and ultimately to transform the lives of its people. It also left a lasting impact on health and medicine. Korean doctors returned as heroes and this naturally boosted their careers. As parasitic infections loomed large in civilian and military activities in Vietnam – as well as in the ROK – those specialising in these areas did particularly well. One can see this not only in the case of military doctors such as Kim but also in the careers of civilians such as Chu, who received an award from the US State Department in 1968 in recognition of his services in Vietnam and, in the following year, from his own government. After Chu returned from Vietnam, he took up a new position as Professor of Parasitology at Kyung Hee University Medical College in Seoul and was to remain there for the rest of his career. He subsequently became chairman of the Korean Parasitology Society and, in 1974, was awarded a prize for his work in preventive medicine from the Korean Society for Medicine.^129^ These achievements were important not only for Chu but for parasitology as a whole. However, we can see from Chu’s subsequent publications that his approach had changed. Although he continued to express concern about the incidence and prevalence of parasitic infection in Korea, his focus was increasingly on treatment rather than on hygiene and education.^130^

In this respect, Chu’s publications were far from unusual. Up to the late 1960s, most articles on parasitic infection were epidemiological studies advocating preventive strategies such as the reform of dietary habits.^131^ But the experience of doctors in Vietnam and Korea showed that education had its limits. It was evident that cultural changes would come slowly, if at all. In the meantime, clinical studies of anti-helminthic drugs – some herbal, most chemical – offered a viable alternative.^132^ Experiments with various anti-helminthic agents began in the early 1960s and by the end of the decade there were several such drugs in use. Some were employed with success in the army and this was soon followed by their extension to civilians. The latter campaign was enabled by the passage of legislation (in 1966) by the ROK’s National Assembly which required the sampling of faeces obtained from schoolchildren on a twice-yearly basis.^133^ Partly as a result of its participation in the Vietnam War, the government now had the necessary financial resources to fund a national campaign. Chemoprophylaxis with compounds such as bithional sulphoxide promised a quick and cost-effective way of doing this.^134^ But fatal reactions to such drugs among some children meant that treatment still needed to be reinforced by educational messages which emphasised the importance of taking medicine regularly at the required dosage.^135^ These initiatives were complemented by local experiments with periodic mass treatment of Koreans of all ages, such as for roundworms and hookworms.^136^ The results were dramatic and parasitic infection fell to nearly a third of 1969 rates by 1974. By the 1980s, it was negligible, although the sampling of school-children continued until 1988.^137^

## 5 Conclusion

Korean doctors and health workers played a prominent role in campaigns against disease in Vietnam but they were unable to repeat the success they had achieved at home. Moreover, unlike the south of the Korean peninsula, Vietnam could not be saved from communism. For ideologically committed doctors such as Chu Jeong-Gyun, this must have been a matter of great regret. And yet, the war left its mark on Korean medicine, as well as on Korean politics. Civilian and military doctors benefited from their participation in a mission vital to the future of the ROK and this was especially true of those who worked on parasitic infections. This applies not only to specialists in parasitology but also to those who grappled with such diseases as epidemiologists and clinicians. Versatility became a virtue as well as a necessity, enabling doctors to remain relevant to a range of public health concerns throughout their careers. Among these, parasitic diseases continued to loom large and formed a thread running through the work of many doctors, especially those involved in preventive medicine. Although parasitic infections were becoming more prominent in the years leading up to the war, the deployment to Vietnam gave greater impetus to their study and enabled the campaign against parasitic infection to garner more support, culminating in a decisive commitment to eradication. Developments at home and in Vietnam were therefore mutually reinforcing: a reminder that histories of war and medicine must remain sensitive to the role of both in society more generally.^138^

In the Korean case, civilian and military concerns were united by the Republic’s relentless drive for modernisation, and modernisation was enabled by, and was also a consequence of, Korea’s importance in the Cold War. Like other developing nations, including its communist neighbour, the ROK received financial and scientific assistance from the superpowers.^139^ But, at this stage, it was the ROK that was the principal actor. Its anti-communist ideology and the ever-present threat from the North served as a powerful incentive to modernisation; in health and medicine, as well as in other areas of policy. This is evident not only at government level but in the careers of doctors like Chu, who considered the fight against disease, ‘ignorance’ and communism in Vietnam to be identical to that in their own country. They hoped that their endeavours would enable Asian peoples to transcend the legacy of colonialism and what they regarded as superstition in their own cultures.

These attitudes had an enduring effect on policy and practice in Korea, if not in Vietnam. The deployment of medical teams to South East Asia was the first of many Korean contributions to international aid. Korean preventive medicine specialists subsequently provided a good deal of technical assistance to developing countries through government institutions and medical charities.^140^ The ROK also began to participate in United Nations peacekeeping missions, sending medical units and other troops to Western Sahara in 1994, for example.^141^ As in Vietnam, these interventions were bolstered by a conviction that Korea, as a formerly colonised nation and a recipient of aid, had a unique role to play in international development, being able to use its own experience to help other nations achieve freedom and prosperity. However, the ROK’s earliest efforts in overseas aid bore little relation to multilateral initiatives in health and development such as those orchestrated by the WHO.^142^ The intervention in Vietnam was a form of instrumental bilateralism, as was all the aid provided under the Free World Assistance Program. It stemmed from the immediate concerns of the conflict: from the attempted ‘pacification’ of Vietnamese civilians and the need to present Korea and other participating nations in a favourable light. It may be for these reasons that the Korean International Cooperation Agency (KOICA) makes no mention of Vietnam in its history of Korean aid projects; such is the controversy that still surrounds the conflict.^143^

While the ideological baggage of the Cold War appears to have been jettisoned, there is no escaping its legacy. At the start of the new millennium, a Korean organisation named Medics for Vietnam and Peace was formed in order to redress what it saw as the wrongs committed by their countrymen over thirty years earlier, particularly the massacre of civilians.^144^ Dentists, later joined by doctors of Korean traditional and Western medicine, worked side by side to assist the Socialist Republic of Vietnam and foster good will between the two countries. It was a situation that Dr Chu could scarcely have envisaged and would still less have approved.

## Footnotes

1.

*Wôlnam Jeonsoenui Hanguk-Gun: Juwôl Hanguk-Gun 3 nyeonganui Baljachwi*[Korean Forces in Vietnam: Three Years in Vietnam] (Seoul: Kwang Myong Printing Company, 1968). For a similar statement of aims, see Joungwon Alexander Kim, ‘Participation in the Vietnam War’, *World Affairs*, 129, 1 (1966), 28–35.

2.
The latter position is taken by Min Yong Lee in ‘The Vietnam War: South Korea’s search for national security’, in Byung-Kook Kim and Ezra F. Vogel (eds), *The Park Chung Hee Era: The Transformation of South Korea* (Cambridge, MA and London: Harvard University Press, 2011), 403–29. Literature emphasising the economic benefits is reviewed in the same chapter.

3.

Park Tae-Gyun , *Beteunam Jeonjaeng* [The Vietnam War] (Seoul: Hangyeore Chulpansa, 2015); Hoenick Kwan, ‘Anatomy of US and South Korean Massacres in the Vietnamese Year of the Monkey, 1968’, *Asia Pacific Journal*, 5, 6 (2007), 1–30; Charles K. Armstrong, ‘America’s Korea, Korea’s Vietnam’, *Critical Asian Studies*, 33, 4 (2001), 527–40.

4.

Yun Cheong-Ro , *Beteunam Jeonjaengui Han-Guk Sahoesa* [A Social History of Korea in the Vietnam War] (Seoul: Pureun Yeoksa, 2015), 306; ‘Allegations of S. Korean Atrocities Arising 40 years after Vietnam War’, *Los Angeles Times*, 16 May 2015, www.latimes.com/world/asia/la-fg-korea-vietnam-20150516-story.html (accessed 3 June 2016).

5.

Bun-Nyeo Kim , ‘Wôlnamjeon Chamjeon Goyeopje Huyujeung Hwanjaui Geongang Sangtaewa Saenghwal Manjokdoe Gwanhan Yeongu’ [Studies on the Health Conditions and the Degree of Life Satisfaction among Veteran Patients who suffered After-Effects from Agent Orange], Master’s thesis: Kyung Hee University, 2002.

6.
Gukjemunje Yeonguso [International Affairs Research Centre], *Wôlnamjeongwa Hangukui Anjeonbojang*[The Vietnam War and National Security] (Seoul: Gukjemunje Yeonguso, 1966), 93.

7.

John P. DiMoia , Reconstructing Bodies: Biomedicine, Health, and Nation-Building in South Korea since 1945 (Stanford, CA: Stanford University Press, 2013), 163–169.

8.
For example, In-Sok Yeo, ‘A History of Malaria in Modern Korea 1876–1945’, *Korean Journal of Medical History*, 20, 1 (2011), 53–82; In-Sok Yeo, ‘U.S. Military Administration’s Malaria Control Activities (1945–48)’, *Korean Journal of Medical History*, 24, 2 (2015), 35–65, http://dx.doi.org/10.13081/kjmh.2015.24.35(accessed 2 August 2015); Jong-Chan Lee, ‘Korea’s Health Care Policy of the Twentieth Century’, *Korean Journal of Medical History*, 8, 2 (1999), 137–46.

9.

Roger Cooter , Surgery and Society in Peace and War: Orthopaedics and the Organization of Modern Medicine, 1880–1948 (London: Macmillan, 1993); Roger Cooter, ‘Medicine and the Goodness of War’, *Canadian Bulletin of Medical History*, 12 (1990), 637–47; Mark Harrison, ‘The Medicalization of War: The Militarization of Medicine’, *Social History of Medicine*, 9 (1996), 267–76; Mark Harrison, ‘Medicine and the Management of Modern Warfare’, *History of Science*, 34 (1996), 379–410.

10.

In-Sok Yeo , ‘Christian Hospitals and their Contribution to Korean Society during the Korean War’, Yonsei Journal of Medical History, 15, 2 (2012), 7–28; Yun-Jae Park, ‘Japan’s Oriental Medicine Policy in Colonial Korea’, *Korean Journal of Medical History*, 17, 1 (2008), 75–86; Yun-Jae Park, ‘The Trend and Prospect of Studies in the Modern History of Medicine in Korea’, *Korean Journal of Medical History*, 19, 1 (2010), 45–68; Dong-Won Shin, ‘The Formation of the Colonial Policy of Medical Care’, *Hanguk Munhwa* [Korean Culture], 30 (2002), 333–70; Dong-Won Shin, ‘The Historical Interpretation of the Formation of the Modern Health Care System in Late Choson’, *Korean Journal of Medical History*, 5, 2 (1996), 155–68.

11.
See Jeong-Ran Kim , ‘The Borderline of “Empire”: Japanese Maritime Quarantine in Busan *c*.1876–1910’, Medical History, 57 (2013), 226–248.2407034710.1017/mdh.2012.104PMC3867849

12.

Jeong-Ran Kim , ‘Malaria and Colonialism in Korea, *c*.1876–*c*.1945’, Social History of Medicine, 29 (2016), 360–383.10.1093/shm/hkv110PMC592739629731545

13.
Yeo, *op. cit.* (note 8), ‘A History of Malaria’; Kim, *ibid*.

14.

Kobayashi Harujirō , ‘Kanzō Jistoma no Kenkyū’ [Research on Liver-Fluke], *Saikingaku Zasshi* [Journal of Bacteriology], 202 (1912), 1–65; Nagai Hitoo, ‘Chōsen ni Okeru Hai Jistoma no Chōsa Gaikyō’ [Overview of Research on Lung-Fluke in Korea], *Gun’idan Zasshi* [Army Medical Journal], 147 (1925), 1149–80.

15.
Japanese efforts amounted to the mobilisation of Korean civilians to catch crabs and other freshwater shell-fish in an attempt to control the spread of lung-flukes. *Ibid*.

16.

Heo Jong , *Banmin Teukwiui Jojikgwa Hwaldon* [The Organisation and Activities of the Select Committee for the Investigation of Pro-Japanese Activists] (Seoul: Seonin, 2003).

17.

Jwa-Seop Shin , ‘The Policy of the United States Army Military Government in Korea toward Public Health and Medicine in Occupied South Korea’, Korean Journal of Medical History, 9, 2 (2000), 212–232.12194170

18.
Yeo, *op. cit.* (note 8), ‘U.S. Military’.

19.

Frank K. Hardner , United States Navy Reserve (USNR), ‘Narrative Report on Activities of Epidemic Disease Control Unit in Korea’, 20 July 1951, 1–2, EF 37-1/M3-2, 1950, BUMED General Correspondence, 1947–51, RG 52, NARA, National Library of Korea.

20.

H.F. Smith , United States Public Health Service Commissioned Corps (USPHS), ‘Report on the Public Health Problems of the Republic of Korea’, 78–80, Federal Security Agency, PHS, 1950, EF 37-1, 1951, BUMED General Correspondence, 1947–51, RG 52, NARA, National Library of Korea.

21.

*Ibid*., 49–50.

22.

*Ibid*., 78.

23.

Doo-Sub Kim , ‘The Demographic Transition in the Korean Peninsula, 1910–90: North and South Korea Compared’, Korea Journal of Population and Development, 23 (1994), 137, 139. The statistics of the Democratic People’s Republic of Korea (DPRK) seem to be more reliable for the period 1949–63 than for later periods, http://www.ncnk.org/resources/publications/Humanitarian-Conf-2009_Suk-Lee_Availibility_Reliability_DPRK_Statistics.pdf (accessed 13 June 2016).12288771

24.
The DPRK death rate in 1960 was 15.3 per 1000. See Kim, *ibid.*, 137.

25.

Phil Whoon Hong and Kenneth M. Scott , ‘A Review of Pulmonary Tuberculosis in Korea and at Severance Hospital’, Yonsei Medical Journal, 2 (1961), 81.

26.
DiMoia, *op. cit.* (note 7), ch. 4. Park’s preference for Western medicine is evident not only from the thrust of his health policies but also the removal, in 1962, of official recognition from courses and qualifications in acupuncture and moxa: see ‘Jeongbuga ggeun’eun chim ddeum ui Maek Minganeseo Itneunda’ [People will continue with the traditions of acupuncture and moxa despite their being cut by the government], *Ohmynews*, 7 September 2003.

27.
Lee, *op. cit.* (note 8).

28.

*Chosun Ilbo*[Chosun Daily], 18 June 2015.

29.

Han-Il Ree , ‘Unstable Vivax Malaria in Korea’, Korean Journal of Parasitology, 38, 3 (2000), 119–138, 121–7; Y.H. Paik and A.C. van der Gugten, ‘Evaluation Report on the Results of the Passive Case Detection Conducted in the Korea Malaria Pre-Eradication Programme during the Period 1960–5’, *Korean Journal of Parasitology*, 4, 1 (1966), 1–9.1291357210.3347/kjp.1966.4.1.1

30.
‘Outline for the Project Review of the Korea-13 Pre-Eradication Programme with Dr H.S. Chung, Minister of Health and Social Affairs on 26 July 1966’, RG 286: Records of the Agency for International Development, 1966, National Library of Korea.

31.
Yeo, *op. cit.* (note 8), ‘U.S. Military’, 35–6.

32.

Junho Jung , Youngin Park and Ock-Joo Kim , ‘A Social History of Ascaris in the 1960s Korea: From a Norm to a Shameful Disease’, Korean Journal of Medical History, 25 (2016), 167–204.2767946710.13081/kjmh.2016.25.167PMC10565070

33.

H.Y. Kang , ‘Studies on the Intestinal Parasites of the Children in Gim Hae Area (the Irrigated Area of the Nak Dong River), especially for Clonorchis sinensis’, Uidae Hakbo [Medical College Journal], 1 (1955), 84–89.

34.
DiMoia, *op. cit.* (note 7), 155–6.

35.

Duk-Jin Yun , ‘Paragonimiasis in Children in Korea: Related to the Custom of Ingesting Raw Crayfish for the Treatment of Measles’, Journal of Pediatrics, 56, 6 (1960), 736–751.1384669810.1016/s0022-3476(60)80310-1

36.

Choong-Moo Park , ‘Epidemiological Aspects of Paragonimiasis in Korea’, Yonsei Medical Journal, 3, 1 (1962), 85–92; ‘Ddo Hanaeu Wiheom Sinho Sae Gisaengchung Anisakiseu’ [Another Danger Signal: A New Parasite Anisakiseu], *Chosun Ilbo*, 18 July 1967, 5.

37.
The study of 14 628 faecal samples taken from outpatients at Severance hospital found an infection rate of 81.7%. See Chin-Thack Soh, Keun-Tae Lee, Eui-Woong Shin, Tae-Chul Kang, ‘Incidence of Parasites in Seoul Area based on an Examination of the Severance Hospital Out-Patients’, *Yonsei Medical Journal*, 2 (1961), 31–47.

38.

*Ibid*., 46.

39.

In-Sok Yeo , ‘A history of public health in Korea’, in M.J. Lewis and K.L. MacPherson (eds), Public Health in Asia and the Pacific: Historical and Comparative Perspectives (London: Routledge, 2008), 82; Hong-Gwan Seo, Sang-Ik Hwang and Jong-Il Chae, ‘Study on the Transition of Intestinal Parasites in Korea from 1913–89’, *Korean Journal of Medical History*, 1 (1992), 45–63.

40.
DiMoia, *op. cit.* (note 7), 3.

41.

Seonsam Na , ‘East Asian Medicine in South Korea’, Harvard Asia Quarterly, 14, 4 (2012), 44–56.

42.

Ayo Wahlberg , ‘A revolutionary movement to bring traditional medicine back to the grassroots level: on the biopoliticization of herbal medicine in Vietnam’, in L. Monnais and H.J. Cook (eds), Global Movements, Local Concerns: Medicine and Health in Southeast Asia (Singapore: NUS Press, 2012), 207–225.

43.

Young-Sun Hong , Cold War Germany, the Third World and the Global Humanitarian Regime (Cambridge: Cambridge University Press, 2015); Trung Dzu, ‘How Great Britain, France, Japan, National China, Malaysia, India and others assist South Vietnam’, *Alliance for Freedom*, 1, 15 July 1968, 10.

44.
Yun, *op. cit.* (note 4), 306; Joo, Hyo Sung, ‘South Korean Men and the Military: The Influence of Conscription on the Political Behavior of South Korean Males’ (2015), *CMC Senior Theses* Paper 1048, http://scholarship.claremont.edu/cmc_theses/104 (accessed 5 June 2016).

45.

S. Koth , B.S. Park and W.T. Longstreth Jr. , ‘The Vietnam War and Medical Research: Untold Legacy of the U.S. Doctor Draft and the NIH “Yellow Berets”’, Academic Medicine, 86, 4 (2011), 502–508.2134649410.1097/ACM.0b013e31820f1ed7

46.
See, for example: Steve Sturdy, ‘War as experiment: physiology, innovation and administration in Britain, 1914–18: the case of chemical warfare’, in R. Cooter, M. Harrison and S. Sturdy (eds), *War, Medicine and Modernity* (Stroud: Sutton, 1999), 65–84; Cooter, *op. cit.* (note 9).

47.
In 1967, US forces in Vietnam experienced hospital admission rates from malaria approaching 10% of troops stationed in Vietnam. In that year, military operations were seriously compromised by the disease and in some units the admission rate was closer to 70%. See Spurgeon Neel, *Medical Support of the US Army in Vietnam 1965–70* (Washington DC: Department of the Army, 1991), ‘Chapter II: Health of the Command’, 34–40,www.history.army.mil/books/Vietnam/MedSpt/chpt2.htm (accessed 8 October 2015).

48.

Doo-Hie Kim , ‘Epidemiological Studies of Malaria in Republic of Korea Forces in Viet-Nam, I: Epidemiological Observation on the Malaria Patients’, Journal of the Korean Medical Association, 10, 9 (1967), 668–678.

49.

*Ibid*., 678.

50.
Brendan O’Keefe with F.B. Smith, *Medicine at War: Medical Aspects of Australia’s involvement in Southeast Asia, 1950–72* (St Leonards, NSW & London: Allen & Unwin, 1994), 148.

51.

Doo-Hie Kim , ‘Epidemiological Studies of Malaria in Republic of Korea Forces in Viet-Nam, II: Observation on the Asymptomatic Malaria’, Journal of the Korean Medical Association, 11, 11 (1968), 901–907; Myeong-Shin Chae, ‘The Characteristics of the Vietnam War, Combined Operations’, *Beteunam Jeonjaeng*, 1 (2002), 1–103, 31.

52.
Kim, *ibid*.

53.

Sam-Sup Choi , ‘A Field Study on Effectiveness of Diamondiphenylsulfone (D.D.S.) as a Suppressive Agent for Chloroquine Resistant *Plasmodium falciparum* Malaria’, Chonnam Medical Journal, 6, 2 (1969), 207–216.

54.
Neel, *op. cit.* (note 47), 39.

55.
O’Keefe, *op. cit.* (note 50), 154–9, 180–7.

56.

Doo-Hie Kim , Dong-Chul Kim , Sang-Bin Park , Kyung-Ho Yang and Kyu-Dong Lee , ‘Epidemiological Studies of Malaria in Republic of Korea Forces in Viet-Nam, III: Observation on the Distribution of Anopheline Larvae’, Kyungpook University Medical Journal, 10, 2 (1969), 199–207.

57.

Gregg Brazinsky , Nation Building in South Korea: Koreans, Americans, and the Making of a Democracy (Chapel Hill, NC: University of North Carolina Press, 2009), 138–140.

58.
‘Juwôl Hanguk Guninui Gamyeom Doen Malariae Daehan YeokhakJeok Yeongu Oe Bunonmun Gupyeon’ [Epidemiological Studies on Malaria among Korean Soldiers in Vietnam and other Papers], PhD thesis: Kyungbuk University, 1969.

59.

Doo-Hie Kim , ‘Epidemiological Studies of Malaria in Republic of Korea Forces in Viet-Nam, IV: Observation on the Concentration of Trophozoites in the Blood Stream in Asymptomatic Malaria’, Journal of the Korean Medical Association, 12, 8 (1970), 655–660.

60.

Sam-Sup Choi , ‘An Epidemiological Study on Malaria in the Republic of Korea Forces, Vietnam’, Chonnam Medical Journal, 6, 2 (1969), 195–205.

61.

Byong-Seol Seo , Soon-Hyung Lee , Jong-June Yoon and Yong-Suk Ryang , ‘Parasitological Studies of Korean Forces in South Vietnam, I: Examination of Blood Films on Malaria Patients’, Korean Journal of Parasitology, 8, 1 (1970), 25–29.1291352210.3347/kjp.1970.8.1.25

62.
‘Gukga Siheom Sangim Isa Choi Sam-Sup ssi’ [Obituary of Choi Sam-Sup], *Yakguk sinmun*[Pharmacy Newspaper], 8 September 2000. Choi became the Vice-President of the National Medical Licensing Board.

63.

Jong-Soo Lee , Wonn-Ik Chang , Tae-Whan Son , Jae-Young Jung and Tae-Kyoo Shin , ‘The Clinical Study of 157 Cases of Malaria Evacuated from Viet Nam’, Journal of the Korean Medical Association, 10, 4 (1967), 318–326; J.S. Lee, T.K. Shin, T.H. Sohn, J.Y. Chung, J.G. Yoo, C.S. Lee, and K.S. Byun, ‘The Clinical Study of 200 Cases of Falciparum Malaria Evacuated from Vietnam’, *Korean Journal of Internal Medicine*, 11, 6 (1968), 23–7.

64.

K.S. Byun , Y.S. Paek , K.S. Lee , W.I. Chang , T.D. Sohn , Y. Park and K.Y. Lee , ‘A Case of Cerebral Malaria Accompanied by Black Water Fever’, Korean Journal of Internal Medicine, 10, 10 (1967), 65–67.

65.

Kee-Yoon Yoon , Jin-Kwan Lee and Myung-Hoon Ahn , ‘Clinical Observations of Drug-resistant Malaria from Vietnam’, Korean Journal of Internal Medicine, 10, 10 (1967), 43–50.

66.
For falciparum and mixed malaria, the best results were usually obtained from oral administration of quinine and pyrimethamine with sulphonamides, whereas for vivax malaria single therapy with chloroquine was most effective: Ki-Soo Byun, Yung-Soo Baek, Kong-Suk Lee, Won-Ick Chang, Yung Park, ‘Clinical Observation on 1000 Cases of Malaria from the Nha-Trang Area in Vietnam’, *Korean Journal of Internal Medicine*, 11, 6 (1968), 363–75; Jeong-Wan Han, Gwang-Hak Lee and Gyu-Dong Lee, ‘Beteunam Eseoui Yeoldae Malariaui Imsang Gyeongheom’ [Clinical Experience of *Plasmodium falciparum*in Vietnam], *Gamyeom* [Infection], 1, 1 (1969), 27–32.

67.
In this sense, it had a similar effect to parasitic infection among labourers who left the ROK to work in West Germany. See Jung, Park and Kim, *op. cit.* (note 32).

68.

Joong-Ho Kim , Jong-June Yoon , Soon-Hyung Lee and Byong-Seol Seo , ‘Parasitological Studies of Korean Forces in South Vietnam, II: A Comparative Study on the Incidences of Intestinal Parasites’, Korean Journal of Parasitology, 8, 1 (1970), 30–35. See also notes 61 and 63, *op. cit.* for the authors’ other papers.1291352310.3347/kjp.1970.8.1.30

69.

Seung-Dae Park , Seung-Doo Kim and Yong-Suk Ryang , ‘The Prevalence of Intestinal Parasites in ROKA Soldiers at Won-Ju Area’, Korean Journal of Parasitology, 7, 3 (1969), 167–170.1291353010.3347/kjp.1969.7.3.167

70.

Kyung-Il Im , Bang-Bu Youn and Suk-Kyoung Lee , ‘The Prevalence of Intestinal Parasites in ROKA Soldiers’, Korean Journal of Parasitology, 10, 1 (1972), 1–7.1291350510.3347/kjp.1972.10.1.1

71.
For example, the epidemiological unit sent to Vietnam by the Ministry of Health and Welfare in 1967. See ‘Isibilgge Yeokhak Josadan Pawôl’ [An Epidemiological Investigation Team will be sent to Vietnam on 20th January], *Chosun Ilbo*, 8 January 1967. For an overview of KOPREM’s activities, see Chu Jeong-Gyun, ‘Wôlnam Bogeonui Hyeonsilgwa Jeonmang’ [The Reality and Prospect of Public Health in Vietnam], *Uisasinbo*[Medical News] (1971), 865, 10; 866, 10. Medical aid was the largest single type of aid delivered under the Free World Assistance Program, constituting 32.5% of all assistance, the next largest being education (24.3%). See USAID, *Free World Assistance to Vietnam: A Summary of the Economic, Technical, Social and Humanitarian Assistance Granted by the Free World Countries to Vietnam July 1, 1964–June 30, 1968*, 3, pdf:usaid.gov/pdf-docs/Pnadl119.pdf (accessed 4 June 2016).

72.

Jung-Kyun Chu , ‘End of Tour Report, 14 August 1967–13 August 1968, 1–2, Chu Collection, Kyung Hee University (KHU).

73.
Public Health Division/CORDS III CTZ, Personnel Status Report, 15 April 1970, Chu Collection, KHU.

74.

Jeong-Gyun Chu , *op. cit.* (note 71), 865, 10.

75.

Jeong-Gyun Chu , Essays in Celebration of His 70th Birthday (Seoul: Gukje Gyoyeok Munhwa, 1989), Preface.

76.

Jung-Kyun Chu and Sung-Moon Hong , ‘Histochemical Studies on Clonorchis sinensis’, Korean Journal of Parasitology, 1, 1 (1963), 11–13; Jung-Kyun Chu and Hyung-Sup Lee, ‘Histochemical Studies on the Relationship between the Ascarides in Man and Pigs’, *ibid*., 15–21.

77.
Epidemiological Intelligence Study, US Communicable Disease Control Service, *c*.1960, Chu Collection, KHU.

78.

Chu Jeong-Gyun , KOPREM Team Report (1968–9), 3, Chu Collection, KHU.

79.

*Ibid*., 2.

80.
Chu did not really distinguish between Chinese and Vietnamese medical traditions, which developed to some extent independently and had many unique features. See C. Michelle Thompson, *Vietnamese Traditional Medicine: A Social History* (Singapore: NUS Press, 2015).

81.

Chu Jeong-Gyun , ‘Wôlnam Pest’ [Plague in South Vietnam], *Uisasinbo* (1968), 591, 9; 592, 9; 593, 7; 618, 9.

82.
Chu, *op. cit.* (note 78), 2–3.

83.

*Ibid*., 3.

84.

*Ibid*., 4.

85.
L. Monnais, C.M. Thompson and A. Wahlberg (eds), *Southern Medicine for Southern People: Vietnamese Medicine in the Making* (Newcastle upon Tyne: Cambridge Scholars Publishing, 2012), 9–10, 12.

86.

Laurence Monnais , ‘“Could confinement be humanised?” A Modern History of Leprosy in Vietnam’, in M.J. Lewis and K.L. MacPherson (eds), Public Health in Asia and the Pacific: Historical and Comparative Perspectives (London: Routledge, 2008), 123–124.

87.

L. Monnais , introduction in Monnais, Thompson and Wahlberg, *op. cit.* (note 85), 11.

88.
Lee Bu-Yeong (ed.), *Uihak Gaeron: Uihakui Gaenyeomgwa Yeoksa*[An Introduction to Western Medicine: The Concept of Medicine and its History] (Seoul: Seoul National University Press, 1994).

89.
Communication to the authors from Dr Chu Jong-Phil (Dr Chu Jeong-Gyun’s son), 10 September 2015.

90.
Joint Preventive Medicine Subcommittee of the Medical Policy and Coordinating Committee [hereafter, JPMS], ‘Disease Control in the Republic of Vietnam: Elements of a Program’, 5, Chu Collection, KHU.

91.
Hong, *op. cit.* (note 43), 118–9.

92.
End of Tour Report, Lt.-Gen. A.S. Collins Jr, Commanding General, I Field Force Vietnam, 23, RG 472, National Library of Korea.

93.

*Ibid*., 3–4.

94.

*Ibid*., 54.

95.
JPMS, *op. cit.* (note 90), 1.

96.

*Ibid*., 1–2.

97.
These activities lie beyond the scope of the present study, but see Choong-Hyun Hwang, ‘The Evaluation of the First Year of National Plague Control in Vietnam’, *Yonsei Reports on Tropical Medicine*, 2, 1 (1971), 101–9; Chu Jyun-Kyun, ‘Tropical Medicine in South Vietnam’, 7–8, Chu Collection, KHU. On the epidemics, see Myron Allukian Jr and Paul L. Atwood, ‘Public health and the Vietnam War’, in B.S. Levy and V.S. Sidel (eds), *War and Public Health* (Washington DC: American Public Health Association, 2000), 215–37.

98.
Chu, *op. cit.* (note 97), 15.

99.
There were no indigenous falciparum cases in Korea other than a few resulting from intravenous contamination but in the 1930s some naturally transmitted cases of ‘quartan fever’ produced by the parasite *Plasmodium malariae*were recorded.

100.
Chu, *op. cit.* (note 97), 15–6.

101.
JPMS, *op. cit.* (90), 2.

102.

*Ibid*., 2.

103.
Chu, *op. cit.* (note 97), 15–8.

104.

*Ibid*., 19.

105.
Chu, *op. cit.* (note 72), 8–9.

106.
JPMS, *op. cit.* (note 90), 1. For the much larger US contribution to civilian medical aid, see Robert J. Wilensky, *Military Medicine to Win Hearts and Minds: Aid to Civilians in the Vietnam War* (Lubbock, TX: Texas Tech University Press, 2004).

107.
JPMS, *op. cit.* (note 90), 3.

108.

*Ibid*., 3.

109.
Chu, *op. cit.* (note 72), 6, 12.

110.

*Ibid*., 4.

111.
Chu, *op. cit.* (note 72), 8.

112.

*Ibid*., 5.

113.

*Ibid*., Appendix, Table 1.

114.
‘Outline for the Project Review of the Korea-13 Pre-Eradication Programme with Dr H.S. Chung, Minister of Health and Social Affairs on 26 July 1966’, RG 286: Records of the Agency for International Development, 1966, National Library of Korea; Paik and Van der Gugten, *op. cit.* (note 29).

115.

Richard Hunt , Pacification: The American Struggle for Vietnam’s Hearts and Minds (Boulder, CO: Westview Press, 2009).

116.
Collins, *op. cit.* (note 92), 23.

117.
Collins, *op. cit.* (note 92), 22.

118.
Park, *op. cit.* (note 3), 143.

119.

Ba-Ngill, ‘R.O.K.-R.V.N. Alliance Against Communism’, Alliance for Freedom, 1(1968), 21.

120.
Park, *op. cit.*, (note 3), 142–55; Gukjemunje Yeonguso, *op. cit.* (note 6), 112–4, 119–20.

121.
Anon, *op. cit.* (note 1), 67.

122.
For example, *Donga Ilbo*[Donga Daily], 17 May 1965, 4; 17 May 1967, 6; 15 May 1968, 4.

123.
For example, ‘Pyeonghwareul Alneun Wôlnam’ [Vietnam is Eager for Peace], *Donga Ilbo*, 24 May 1967, 6; 26 July 1968, 7.

124.
‘Kollera Juuibo’ [Cholera Alert], *Donga Ilbo*, 13 August 1968, 5.

125.
‘Gosanjok Ggaeuchineun Ddaihan Insul’ [Civilising Villagers in Isolated Areas using Korean Medical Treatment], *Chosun Ilbo*, 21 September 1967, 6.

126.
‘Wôlname Simeun Ddaihan Insul [Korean Medical Treatment implanted in Vietnam], *Chosun Ilbo*, 12 October 1967, 4.

127.
‘Jakjeom Suhaenge Nanjeon’ [Military Operational Difficulties], *Donga Ilbo*, 11 August 1968, 4.

128.

*Donga Ilbo*, 22 August 1969, 4.

129.
Chu, *op. cit.* (note 75), preface.

130.

Jung-Kyun Chu and Jung-Cho You , ‘Studies on Efficiency and Dosage for Schoolchildren of Some Antihelminthic Drugs’, Korean Journal of Parasitology, 10, 1 (1972), 147–156.10.3347/kjp.1974.12.2.14712913477

131.

J.T. Lee , ‘Studies on the Metacercariae from Fresh Water Fishes in the Kum-Ho River’, Korean Journal of Parasitology, 6, 3 (1968), 76–100; M.S. Kim and I.K. Loh, ‘Survey on the Eating Habits of Koreans and Parasitic Infection in Seoul City, Korea’, conference abstract, *Korean Journal of Parasitology*, 6, 2 (1968), 19.10.3347/kjp.1968.6.3.7712913543

132.

Hyung-Soon Lee , Se-Chul Kang , Jong-Ho Ahn , Jung-Woon Lee and Han-Jong Rim , ‘Santonin-Kainic Acid Complex as a Mass Chemotherapeutic of *Ascaris lumbricoides* Control in Korea’, Korean Journal of Parasitology, 10, 2 (1972), 79–85; Chae-Joo Park, Taesic Park and Chong-Won Kim, ‘Development of Herbal Antihelminthics in Korea’, *Report of NIH Korea* (1970), 187–9.1291350010.3347/kjp.1972.10.2.79

133.
Seo, Hwang and Chae, *op. cit.* (note 39), 49; DiMoia, *op. cit.* (note 7), ch. 5.

134.

Il Chung , ‘Trematode Infection in Korea’, Official Journal of the Research Institute of Medical Science of Korea, 2, 5 (1970), 33–40.

135.
‘Hoichungyak Meokgo Jeolmyeong’ [Sudden Death after taking Antihelminthic Drugs], *Chosun Ilbo*, 9 April 1967, 7.

136.

Dong-Chan Kim , ‘Studies on the Control of Ascaris and Hookworm Infection by Periodic Mass Treatment: 1st Year Report’, *Report of NIH Korea* (1970), 281–306; Yeo, *op. cit.* (note 39), 82.

137.
Seo, Hwang and Chae, *op. cit.* (note 39), 53.

138.

Roger Cooter and Steve Sturdy , ‘Of war, medicine and modernity’, introduction in Cooter, Harrison and Sturdy (eds), *op. cit.* (note 46), 7.

139.
See Marcos Cueto, *Cold War, Deadly Fevers: Malaria Eradication in Mexico, 1955–75* (Baltimore, MD: Johns Hopkins University Press, 2007); Sanjoy Bhattacharya, *Expunging Variola: The Control and Eradication of Smallpox in India, 1947–77* (Hyderabad: Orient Longman, 2006); Randall M. Packard, ‘Malaria Dreams: Postwar Visions of Health and Development’, *Medical Anthropology*, 17 (1997), 279–96; Randall M. Packard, *A History of Global Health: Interventions into the Lives of Other Peoples* (Baltimore, MD: Johns Hopkins University Press, 2016), chs 6 and 8.

140.
Yeo, *op. cit.* (note 39).

141.
See Kim Jeong-Hee, ‘Seobu Sahara Gukgun Uiryo Jiwôn danwônui Seuteureseu Yoine Gwanhan Yeongu’ [Causes of Stress in the Korean Military Medical Unit in Western Sahara], Master’s thesis: Yonsei University Department of Public Health, 2001).

142.
See, for example, Sunil S. Amrith, *Decolonizing International Health: India and Southeast Asia, 1930–65* (Basingstoke and New York: Palgrave Macmillan, 2006); Amy L.S. Staples, *The Birth of Development: How the World Bank, Food and Agriculture Organization, and World Health Organization Changed the World, 1945–65* (Kent, OH: Kent State University Press, 2006); Kelley Lee, *The World Health Organization* (Abingdon: Routledge, 2009).

143.
See http://www.koica.go.kr/english/koica/koica_glance/history/index.html(accessed 2 October 2015).

144.

Lee Seon-Yeong , ‘Beteunam Pyeonghwa Uiryoyeondae’ui Sipnyeon Hwaldonggwa Jeonmang’ [Ten Years’ Work by Medics for Vietnam and Peace, and Future Prospects], Master’s thesis: Songgonghoe University, 2011.

